# Cervical lymphadenopathy – an unusual presentation of carcinoma of the cervix: a case report

**DOI:** 10.1186/1752-1947-2-252

**Published:** 2008-07-28

**Authors:** Madhavi Manoharan, Durga Satyanarayana, Arjun R Jeyarajah

**Affiliations:** 1 St Bartholomew's Hospital, London, UK; 2 17, Benhurst Avenue, Elm Park, Hornchurch, Essex, RM12 4QS, UK

## Abstract

**Introduction:**

The clinical presentation of carcinoma of the cervix as cervical lymphadenopathy has not been described before. We report a case of this unusual manifestation of cervical cancer.

**Case presentation:**

A 51-year-old woman presented to our Head and Neck department with cervical lymphadenopathy. A positron emission tomography scan revealed the primary tumour to be in the cervix and a cervical biopsy confirmed carcinoma of the cervix.

**Conclusion:**

Recurrences of carcinoma of the cervix presenting as lymphadenopathy have been described before but this is the first time a clinical presentation of carcinoma of the cervix as cervical lymphadenopathy has been described. Although metastasis from the cervix to the cervical lymph nodes is rare, this can be explained by outlining the drainage of the lymphatic system from the cervix.

## Introduction

Carcinoma of the cervix commonly metastasizes by direct extension or lymphatic dissemination within the pelvis. Clinical presentation of carcinoma of the cervix as cervical lymphadenopathy has not been described before. We report a case of this unusual manifestation of cervical cancer.

## Case presentation

A 51-year-old woman was referred to the ENT department with a 2-week history of a lump on the right side of her neck. There was no history of change to her voice or dysphagia.

She is a para 4 with all normal vaginal deliveries and has had normal cervical smears in the past. Her periods were regular and she gave no history of intermenstrual or post-coital bleeding. She smoked about 20–30 cigarettes per day.

On further questioning in the clinic, she gave a history of increasing lethargy for the past 3 months and was also unable to report to work due to severe back pain.

Five years before the present episode, she reported feeling unwell with significant weight loss and heavy periods. She was found to be anaemic and was given five units of blood. She was investigated for a possible colon cancer which proved to be negative. She was referred to a Menstrual Disorder Clinic but failed to attend the clinic twice.

On examination, multiple cervical lymph nodes were palpable on both sides of the neck. Ultrasound scan of the neck revealed two large supraclavicular lymph nodes with several abnormal looking lymph nodes in the right carotid chain.

An X-ray of the chest showed no abnormality. Fine needle aspiration of the lymph nodes yielded squamous carcinoma cells.

Metastatic squamous cell carcinoma of an unknown primary tumour was suspected and investigations were performed to find a possible primary site. Clinical examination and endoscopy of the upper digestive tract did not yield an obvious primary tumour in the nasopharynx, larynx and hypopharynx.

Computerised Tomography (CT) of the neck, chest and abdomen revealed marked mediastinal and para-aortic lymphadenopathy suggestive of spread of the known squamous cell carcinoma. There was evidence of dilatation of the collecting system bilaterally with dilatation of the proximal ureters suggesting an obstruction within the pelvis.

**Figure 1:**
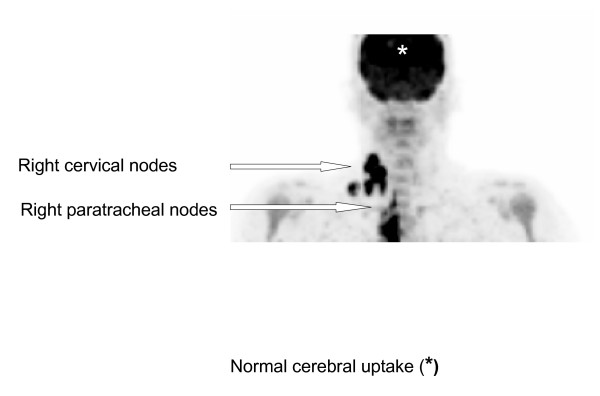
Coronal PET image of FDG uptake in the head and neck.

**Figure 2:**
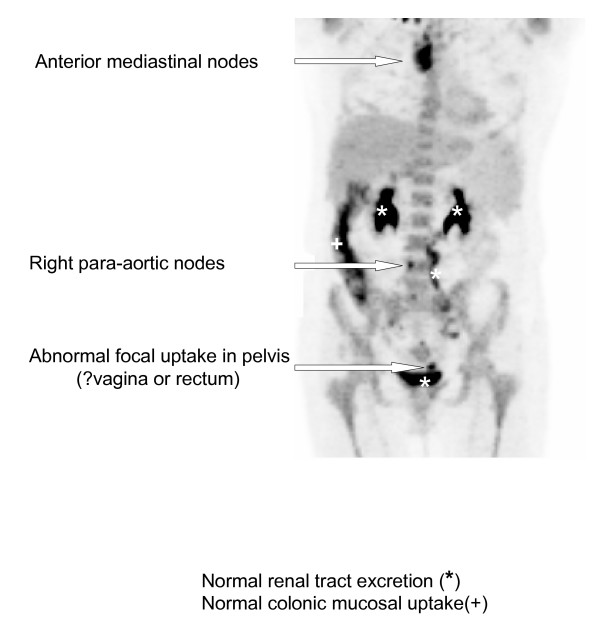
**Coronal PET image of FDG uptake and excretion in the chest, abdomen and pelvis**.

A Positron Emission Tomography-CT (PET-CT) scan was performed which showed markedly increased uptake in the right cervical lymph nodes, as well as in the right paratracheal, anterior mediastinal, lower para-aortic, and bilateral iliac lymph nodes with an obturator node showing a photopaenic centre. In addition, there was a focal area of increased uptake in the pelvis, suggesting a lesion within the rectal wall or in the vaginal vault (Figures 1 and 2).

Given the histology of squamous carcinoma, the PET scan suggested that the uptake in the pelvis may represent a primary gynaecological problem rather than a second malignancy in the rectum. But given the distribution of the disease which was very unusual for cervical carcinoma, a review of the histology was suggested with a differential diagnosis of lymphoma to be considered. The histology from fine needle aspiration of the cervical lymph node confirmed it to be carcinoma cells of squamous origin.

Our patient was then referred to the gynae-oncology team. On examination, the uterus was anteverted, mobile and bulky corresponding to about 14 weeks' size with no palpable adnexal masses. Her cervix appeared normal to the naked eye and a smear was obtained which was reported as normal.

**Figure 3:**
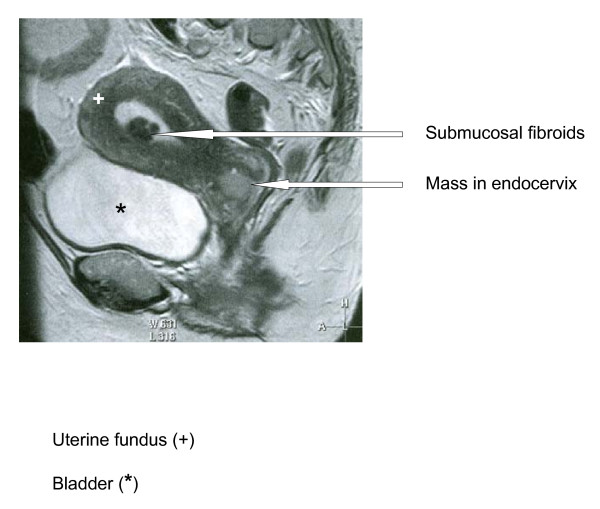
**Sagittal T2-weighted MR image through the midline of the pelvis**.

Magnetic Resonance Imaging (MRI) of the pelvis and abdomen was performed which revealed a highly abnormal cervix, diffusely infiltrated by an intermediate to high T2 signal intensity mass measuring approximately 3 × 4 × 3.5 cm. The mass involved the endocervical canal and the stroma with suspected early parametrial invasion anteriorly. There was no convincing evidence to suggest bladder involvement and the rectum was clear of disease. Several small intramural fibroids were demonstrated within the myometrium as well as a submucosal fibroid in the anterior body of the uterus (Figure 3).

There was extensive lymphadenopathy along both pelvic side walls, common iliac regions and the para-aortic regions but with no evidence of inguinal lymphadenopathy. Bilateral hydronephrosis was noted. No bony deposit was seen. In conclusion, the MRI reported that the appearance was consistent with a cervical carcinoma with extensive lymphadenopathy and hydronephrosis, stage FIGO 3b.

Routine blood investigations before examination under anaesthesia showed her to be anaemic with a haemoglobin level of 6 g/dl. She was transfused with four units of blood. Her liver function tests and renal function tests were normal and serology showed her to be negative for HIV.

She had an examination under anaesthesia, cervical biopsy and an endocervical and endometrial curettage. Examination under anaesthesia showed the cervix to be bulky with an intact surface epithelium. There was no parametrial involvement and the rectum and bladder were free. Hysteroscopy revealed a pedunculated fibroid on the anterior wall of the uterus. Large biopsies of the anterior and posterior lip of the cervix were taken which identified a poorly differentiated squamous cell carcinoma of the anterior lip of the cervix. The endocervical curettings were positive for squamous cell carcinoma and the endometrial curettings showed proliferative phase endometrium.

With an impression of metastatic squamous cell carcinoma of the cervix, she was started on palliative chemotherapy with carboplatin and paclitaxel. She has responded well to the therapy with a reported decrease in the size of the neck nodes.

## Discussion

In the case of carcinoma of the cervix, metastasis to the neck signals a grave prognosis for the patient. Although very uncommon, spread of carcinoma from the uterine cervix to the supraclavicular region is best understood through a description of the lymphatic system. Carcinoma of the cervix uteri spreads by lymphatics from the pelvis up to the para-aortic nodes, into the mediastinum and then into the thoracic duct. Spread can occur from the pelvis into the hepatic region through the diaphragm and the thoracic duct. The thoracic duct communicates with the central venous system in the neck at the junction of the left subclavian and internal jugular vein. The left-sided supraclavicular node represents the final common path of the body's infra-diaphragmatic lymphatic drainage [1]. Small communications exist from the left side to the right side of the neck.

On reaching the lymph nodes, the embolus of tumour cells begins to multiply, and penetrates the subcapsular tissue leading to local spread into the region surrounding the lymph node. Blockage of the lymph nodes leads to retrograde spread of tumour. This would account for spread from the left side to the right side of the neck, even though there is no direct connection to the right side.

In Henriksen's study [2], incidence of metastasis of carcinoma of the cervix to left supraclavicular nodes was 0.1% in untreated patients but up to 1.5% in treated patients. As further recent studies have shown, modern radiotherapy achieves better control of cancer in the pelvis and allows more patients to survive longer, which, in turn, permits distant metastases to become clinically evident. Hilar, mediastinal [3,4] and supraclavicular lymphadenopathy [5] have been described as the first evidence of tumour recurrence.

But the first presentation of cancer of the cervix with distant metastases in the supraclavicular nodes with a normal looking cervix has not been described before.

The eventual diagnosis of cervical cancer in our patient has been difficult. When she first presented to the Head and Neck department, diagnostic work-up for cervical metastases from an unknown primary was done. As part of this intensive work-up, a (18)F-fluorodeoxyglucose positron emission tomography with computed tomography (FDG-PET-CT) was done, which surprisingly suggested the possibility of a primary in the cervix.

PET is a functional diagnostic imaging technique and has the advantage of being non-invasive and able to study the biological function of the tumour. Increased glucose metabolism has been observed in tumours [6] and F-18 fluoro-2-deoxy-d-glucose (FDG) is a commonly used radiopharmaceutical and is an analogue of glucose [7].

Guntinas-Lichius et al. [8] have shown FDG-PET to have the best sensitivity of 69% and the highest negative predictive value of 87% in detecting unknown primary tumours.

Other studies have shown FDG-PET to have a sensitivity of 100% and sensitivity of 94% in the detection of unknown primary tumours. For the conventional diagnostic modalities (CT and/or MRI, panendoscopy), these values were 92% and 76% [9].

On retrospective review of her past history, her admissions and blood transfusions for anaemia could have been related to underlying cancer of the cervix. But since she did not keep her appointments with the gynaecology clinics, that window of opportunity was lost.

In keeping with her past history, investigation by the gynae-oncology team soon after the CT scan (which had suggested extensive lymphadenopathy) for a possible cervical cause for the lymphadenopathy, would have probably been more cost effective. Due to limited availability and higher cost of the PET scan, it is not routinely used as a primary tool of evaluation. A more thorough work-up and use of other less expensive modalities would have shown the primary to be in the cervix.

It is very unusual for squamous cell carcinoma of the cervix to behave in an aggressive way with metastasis to extrapelvic lymph nodes. Small cell cancer of the cervix is known to be aggressive with early haematogenous and extrapelvic lymph node metastasis [10].

The prognosis for metastatic carcinoma of the cervix is poor. Metastases to the neck signal a grave prognosis for the patient. Diddle [5], in his retrospective review of 18 cases of cervical cancer with metastases to supraclavicular nodes, has quoted a survival time of between 1 and 16 months after the appearance of metastases.

If left alone, cervical nodes grow rapidly with the attendant sequelae of ulceration and pain, making treatment difficult or impossible. Treatment is usually with local irradiation [11].

Treatment of advanced cervical cancer is usually palliative. Several chemotherapy regimes have been described. Cisplatin has emerged as the most active single agent with overall response rates of 19% [12]. Recent phase III trials have documented response rates of 27% and 39% when cisplatin was combined with either paclitaxel or topotecan, respectively [12].

The comparison of cisplatin to cisplatin plus topotecan in GOG-179 has shown a statistically significant impact on the overall response rate, median progression-free survival, and median survival, with all outcome measures favouring the two-drug regimen [13].

Our patient is presently undergoing palliative chemotherapy with a combination of carboplatin and paclitaxel. Her initial response has been encouraging with an anticipated improvement in quality-of-life scores.

## Conclusion

Recurrences of carcinoma of the cervix presenting as lymphadenopathy have been described before but this is the first time a clinical presentation of carcinoma of the cervix as cervical lymphadenopathy has been described.

Although metastasis to the cervical lymph nodes is rare, this can be explained by outlining the drainage of the lymphatic system from the cervix. Prognosis in such patients is usually poor and treatment is mainly palliative.

Although the management of our patient has not changed, this case report highlights an unusual presentation of carcinoma of the cervix and the investigative modalities which were needed to reach the final diagnosis.

## Abbreviations

ENT: Ear, Nose and Throat; CT: Computerised tomography; PET: Positron emission tomography; MRI: Magnetic resonance imaging; FIGO: International Federation of Gynaecology and Obstetrics; HIV: human immunodeficiency virus; FDG: fluorodeoxyglucose.

## Competing interests

The authors declare that they have no competing interests.

## Authors' contributions

MM: Literature review, conceived and drafted the manuscript, DS, Helped in collecting records and preparing the manuscript, AJ, Department chair who provided general support. All authors revised and approved the final draft of the manuscript.

## Consent

Written consent was obtained from the patient for publication of the case report and any accompanying images. A copy of the written consent is available for review by the Editor-in-Chief of this journal.
